# Differential Post-Exercise Blood Pressure Responses between Blacks and Caucasians

**DOI:** 10.1371/journal.pone.0153445

**Published:** 2016-04-13

**Authors:** Huimin Yan, Michael A. Behun, Marc D. Cook, Sushant M. Ranadive, Abbi D. Lane-Cordova, Rebecca M. Kappus, Jeffrey A. Woods, Kenneth R. Wilund, Tracy Baynard, John R. Halliwill, Bo Fernhall

**Affiliations:** 1 Department of Kinesiology and Community health, University of Illinois at Urbana-Champaign, Champaign, IL, United States of America; 2 Department of Kinesiology, East Carolina University, Greenville, NC, United States of America; 3 Department of Kinesiology and Nutrition, University of Illinois at Chicago, Chicago, IL, United States of America; 4 Department of Human Physiology, University of Oregon, Eugene, OR, United States of America; University of Northampton, UNITED KINGDOM

## Abstract

Post-exercise hypotension (PEH) is widely observed in Caucasians (CA) and is associated with histamine receptors 1- and 2- (H1R and H2R) mediated post-exercise vasodilation. However, it appears that blacks (BL) may not exhibit PEH following aerobic exercise. Hence, this study sought to determine the extent to which BL develop PEH, and the contribution of histamine receptors to PEH (or lack thereof) in this population. Forty-nine (22 BL, 27 CA) young and healthy subjects completed the study. Subjects were randomly assigned to take either a combined H1R and H2R antagonist (fexofenadine and ranitidine) or a control placebo. Supine blood pressure (BP), cardiac output and peripheral vascular resistance measurements were obtained at baseline, as well as at 30 min, 60 min and 90 min after 45 min of treadmill exercise at 70% heart rate reserve. Exercise increased diastolic BP in young BL but not in CA. Post-exercise diastolic BP was also elevated in BL after exercise with histamine receptor blockade. Moreover, H1R and H2R blockade elicited differential responses in stroke volume between BL and CA at rest, and the difference remained following exercise. Our findings show differential BP responses following exercise in BL and CA, and a potential role of histamine receptors in mediating basal and post-exercise stroke volume in BL. The heightened BP and vascular responses to exercise stimulus is consistent with the greater CVD risk in BL.

## Introduction

Blacks (BL) are at greater risk for developing hypertension, cardiovascular disease, stroke and renal disease than Caucasians (CA) [1]. Even young, apparently healthy BL, who exhibit comparable blood pressure (BP) to their CA counterparts, have greater macro and microvascular dysfunction [2, 3]. BL have also been shown to exhibit cardiovascular hyper-reactivity to stress with an exaggerated blood pressure response to both behavioral and physiological sympathoexcitation [4].

An acute bout of moderate aerobic exercise causes a sustained reduction in BP in CA, termed post-exercise hypotension (PEH). PEH is usually due to a reduction in peripheral vascular resistance that is not completely offset by a rise in cardiac output [5, 6]. Histamine receptors 1 and 2 (H1R and H2R) have been shown to be responsible for post-exercise vasodilation and associated PEH in normotensive CA [7], but the role of H1R and H2R has not been examined either at rest or during post-exercise recovery in BL.

PEH has been widely observed in both normotensive [8] and hypertensive [9] CA men and women, with greater and more prolonged responses in hypertensives (-2/-3 and -9/-9 mmHg in normotensive and hypertensive individuals, respectively) [10–12]. The prolonged hypotensive effect of regular exercise training in CA may be due to repeated instances of PEH [13, 14]. Thus, understanding the mechanisms of post-exercise BP modulation can serve as a model for understanding the effect of exercise and physical activity on BP. Little information is available on PEH in BL, but some evidence suggests PEH may be absent in this population [15, 16], consistent with their higher risk for hypertension. However, the lack of PEH in those studies may have been a function of diurnal variation [17, 18], or lack of an adequate exercise stimulus.

The aim of the present study was to determine the extent to which normotensive BL develop PEH, and which mechanisms contribute to PEH (or lack thereof) in this population performed during a standardized time of day to avoid diurnal variations. Examining these mechanisms will provide basis for further investigations in clinical populations such as hypertensive patients. The central hypothesis was that PEH would be absent in normotensive BL, but present in CA, and that the lack of PEH in BL would be accounted for by reduced peripheral vasodilation.

## Methods

### Participants

This study was approved by the University of Illinois at Urbana-Champaign Institutional Review Board and each subject gave informed, written consent before participation. Fifty-nine young (age range 18–33 yr), healthy individuals volunteered for this study and signed informed consent.

All participants were free of cardiovascular, metabolic, renal or respiratory disease, and all were non-smokers. Subjects did not take any medications, including over-the-counter pain/anti-inflammatory medication or H1R and H2R antagonists. Volunteers were self-defined as BL or CA if they reported that both parents were of African descent or both parents were of European descent. Female participants had negative pregnancy tests on study visits. All subjects were recruited from the local community or university population.

### Study design

**Fig 1:**
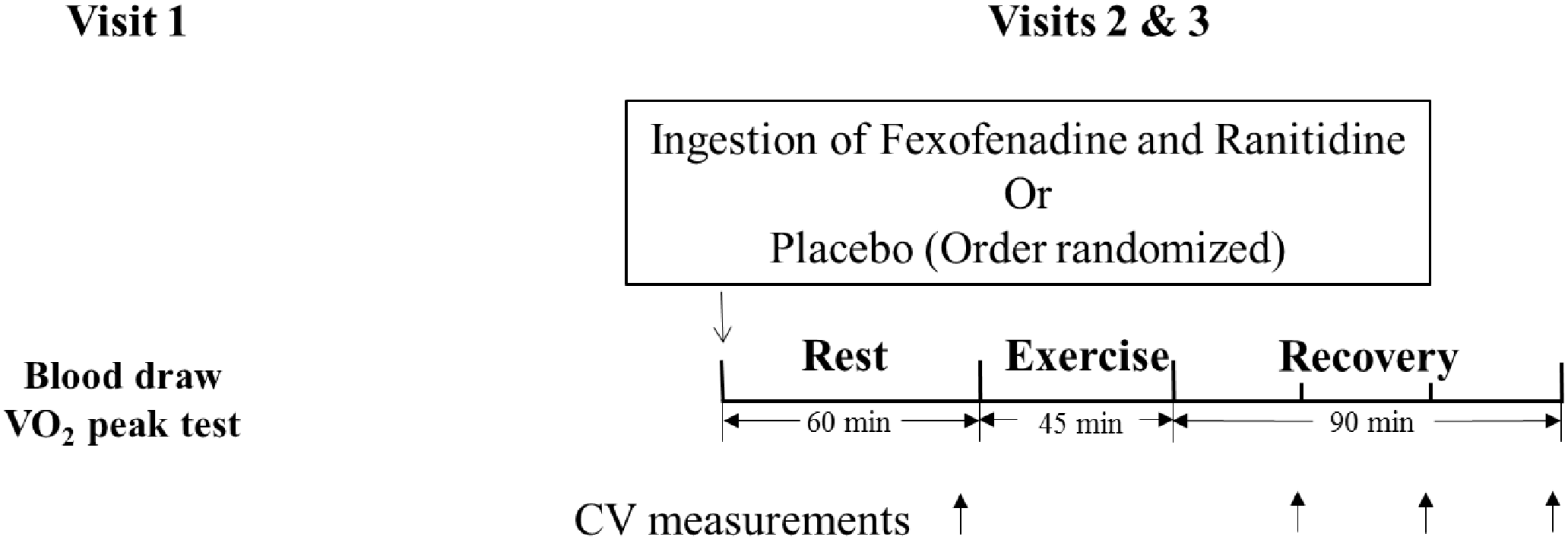
A schematic of the study design showing the procedures for each visit. Subjects underwent testing to determine peak oxygen uptake (VO_2peak_) and a blood draw prior to randomization to placebo or histamine blockade (Visit 1). The order in which the blockade/placebo (visits 2 or 3) were administered was randomized. During study visits 2 and 3, cardiovascular (CV) measurements were obtained at rest and at 30 min, 60 min and 90 min post-exercise (during recovery).

This study employed a randomized, double-blind, counterbalanced design to test the effect of H1R and H2R blockade on PEH. A schematic of the study design is presented in Fig 1. During visit 1, subjects underwent testing to determine peak oxygen uptake (VO_2peak_) and a blood draw prior to randomization to placebo or histamine blockade. During blood draw visits, venous blood samples were obtained after an overnight fast (minimum 12 h fasting). On study visits 2 and 3, participants were given water with either a combined H1R- and H2R antagonist (fexofenadine and ranitidine) or a control placebo (lactose capsules) [19], in a randomly determined order. Prior to each testing session all subjects were asked to fast for minimum of 3 hours. Subjects were also asked to abstain from caffeine, alcohol and exercise for at least 24 hours before each testing session. Men were tested at least 5 days apart between study visits 2 and 3. Women were tested during consecutive early follicular phases (self-report, days 1–5) of their menstrual cycle or placebo phases of the oral contraceptive cycle to control for the influence of hormone fluctuations (visits approximately 30 days apart). The time between study visits 2 and 3 provided more than adequate time for clearance of fexofenadine (half life ~ 12 h) [20] and ranitidine (half life ~ 2.6 h) [21]. The parallel study visits 2 and 3 were made between 3 pm to 8 pm (since exercise-induced hypotension is most consistently observed at this time [18, 22]). During study visits 2 and 3, participants rested in the supine position for 10 min in a quiet and temperature-controlled (22–25°C) room, and then baseline (PRE) BP, cardiac output and peripheral resistance were assessed. After baseline measurements, volunteers exercised on the treadmill for 45 min at 70% heart rate reserve (HRreserve) determined from the VO_2peak_ visit. Cardiovascular measurements were obtained again at 30 min (P30), 60 min (P60) and 90 min (P90) post-exercise with participants in a supine position.

### H1R and H2R blockade

H1R were blocked with 540 mg fexofenadine and H2R were blocked with 300 mg ranitidine. These amounts of fexofenadine and ranitidine have been shown to adequately block H1R and H2R, respectively [20, 21]. Oral ingestion of a H1R antagonist (540 mg of fexofenadine) or H2R antagonist (300 mg of ranitidine) does not cause non-specific cardiovascular effects in the absence of an exercise stimulus. When these drugs were given under normal resting conditions, cardiac output, heart rate, blood pressure, leg blood flow and skin blood flow did not change in CA [23, 24].

### VO_2_peak testing

VO_2peak_ was determined using an incremental graded treadmill exercise test to exhaustion following the Bruce protocol. Heart rate was measured with a Polar Heart Rate Monitor (Polar Electro Oy, Oulu, Finland). Maximal heart rate (HRmax) for each participant was recorded. Expired air was analyzed with a Quark b_2_ breath-by-breath metabolic system (Cosmed, Rome, Italy). The test was terminated when subjects met three of the following five criteria: (1) a final rating of perceived exertion (RPE) score of 17 or greater on the Borg scale (scale 6–20), (2) a respiratory exchange ratio greater than 1.1, (3) no change in heart rate with a change in workload, (4) a “plateau” (increase of no more than 150 ml) in oxygen uptake with an increase in workload, (5) volitional fatigue, defined as an inability to keep up with the treadmill speed.

### Fasting blood measures

All blood draws were carried out in the morning with subjects in a fasted state for at least 12 hours. Blood for lipid profile and glucose was collected in a sterile Ethylenediaminetetraacetic acid (EDTA) vacutainer tubes and was mixed thoroughly by 3 to 6 end-over-end tube inversions to ensure adequate mixing of test sample with anticoagulant. Blood for renal function assessment was collected in a sterile serum separator tube (SST) and the whole blood sample was allowed to clot for 20 minutes and then centrifuged at 2000 × gravitational units (g) for 15 minutes to separate the serum. Both sample tubes were immediately sent out for measurement of the appropriate variables (LabCorp, Champaign, IL). Fasting glucose was assessed via an oxygen rate method using a Beckman Coulter oxygen electrode (Beckman Coulter, Villapointe, France). Total cholesterol, high density lipoprotein cholesterol (HDL cholesterol), and triglycerides (TG) were measured using enzymatic techniques. Low density lipoprotein cholesterol (LDL cholesterol) was calculated using the Friedewald formula. Very low density lipoprotein cholesterol (VLDL cholesterol) was calculated by dividing triglycerides by 5.

### Renal function assessment

Given known racial differences in renal function, estimated glomerular filtration rate (eGFR) was estimated from serum creatinine (sCR) in accordance with recommendations from the Laboratory Working Group of the National Kidney Disease Education Program [25]. eGFR was estimated from the Modification of Diet in Renal Disease Study formula [25].

### Anthropometrics

Anthropometric measurements of body weight and height were made, and body mass index (BMI) was calculated.

### Blood pressure assessment

Following 5 minutes of quiet supine rest in a dimly lit room, resting systolic blood pressure (Brachial SBP) and diastolic blood pressure (Brachial DBP) were measured with an automated oscillometric cuff following established guidelines [26]. All BP measurements were repeated and the average of the two values was recorded and used for analysis. If the values differed by ≥ 5mmHg, a third measurement was obtained and the two closest values were averaged. Brachial mean arterial pressure (Brachial MAP) was calculated as (1/3 * SBP) + (2/3 * DBP).

### Leg blood flow

The diameter of the femoral artery was determined from digital B-mode ultrasound images (Aloka, Alpha 10, Tokyo, Japan) while the ultrasound probe was placed on the skin surface 2–3 cm proximal to the bifurcation of the femoral artery. The mean diameter (D_mean_) was calculated from maximal diameter (D_max_) and minimal diameter (D_min_) weighted to the percentage of time spent at each diameter in the cardiac cycle: D_mean_ = (1/3)D_max_ + (2/3)D_min_. The mean blood velocity (MBV) in the femoral artery was measured using pulse Doppler ultrasonography (Aloka, Alpha 10, Tokyo, Japan) and was taken immediately after diameter measurements.

Mean limb blood flow (Q) was calculated from the mean velocity and area using the formula: Q (ml*min^-1^) = MBVπ r^2^60, where MBV is the mean velocity of the blood (cm*s^-1^), r is the mean radius of the artery during the cardiac cycle (cm), and 60 is a constant (s*min^-1^) to convert the calculated flow from milliliters per second to milliliters per minute. The mean radius of the artery was calculated from the diameter assuming the artery is circular (r = D_mean_/2). This value was doubled to represent both legs. Femoral vascular conductance (FVC) was calculated as flow for both legs/brachial MAP and expressed as ml*min^-1^*mmHg^-1^.

### Cardiac echocardiography

Cardiac output (CO) and stroke volume (SV) were assessed using two-dimensional echocardiography via ultrasound (Aloka, Alpha 10, Tokyo, Japan). With subjects in the left lateral position, measurements were obtained using the four-chamber apical view. The interior endocardial border of the left ventricle was manually traced during both end systole and end diastole. Volumes were measured using Simpson’s rule. SV was calculated by subtracting end systolic volume from end diastolic volume. CO was calculated as HR multiplied by SV. Systemic vascular conductance (SVC) was calculated as CO/brachial MAP and expressed as ml*min^-1^*mmHg^-1^.

### Exercise protocol

Participants were encouraged to hydrate normally before arriving for testing. Exercise was performed in a temperature-controlled room (22–25°C) and water was allowed ad libitum. The aerobic exercise protocol required subjects to exercise continuously for 45 min at 70% HRreserve, which has consistently been shown to cause PEH [9, 27]. Up to 10 min of warm-up was employed to ensure subjects reached their target HR. Subjects were asked to keep their HR at target HR±5 bpm during exercise. HR and RPE were recorded every 10 min during exercise and average HR (ave_HRex) and average RPE (ave_RPEex) were calculated. Water intake during exercise (water_ex) and during the study visit (water_total) was measured. Body weight was measured at the end of the study visit to determine fluid loss.

### Statistical analysis

All data is presented as mean ± SE. Descriptive variables and baseline hemodynamic variables were analyzed with *t*-tests for possible racial differences. Three-way repeated-measures analysis of variance (ANOVA) was used to test for possible condition, race, time and their interaction effects. Post-hoc *t*-tests were conducted if the initial ANOVA yielded significance. We also conducted these probes using ANCOVAs controlling for VO_2peak_. However, using ANCOVA adjusting for cardiorespiratory fitness did not alter statistical results, therefore only unadjusted data from ANOVA analyses was reported. Statistical significance was set at 0.05. SPSS 17.0 (SPSS Inc., Chicago, IL, USA) was used for all analyses.

## Results

### Subject characteristics

Of the 59 subjects initially recruited, 49 (9 BL men, 13 BL women, 14 CA men, and 13 CA women) subjects completed the study. Ten subjects (3 BL men, 3 BL women, 2 CA men, and 2 CA women) dropped out of the study following the initial visit because they were unwilling to continue.

Subject characteristics are shown in Table 1. VO_2peak_ was significantly lower in BL compared to CA (p<0.05) and eGFR was significantly higher in BL compared to CA (p<0.05). There were no significant differences between BL and CA in other anthropometrics or fasting blood measures. Almost all of the subjects were recreationally active (except 1 BL male) with average exercise time between 1–12 hours per week.

**Table 1:** Subject characteristics.

	BL (n = 22)	CA (n = 27)
Age (yrs)	21 ± 1	23 ± 1
Height (cm)	170.9 ± 2.1	172.1 ± 1.9
Weight (kg)	73.4 ± 3.0	72.4 ± 2.7
BMI (kg/m^2^)	25.1 ± 0.8	24.2 ± 0.5
VO_2peak_ (ml/kg/min) ‡	40.0 ± 1.5	45.6 ± 1.2
HRmax (bpm)	192 ± 2	190 ± 2
70%HRreserve (bpm)	154 ± 1	151 ± 1
eGFR (ml.min ^-1^.1.73 m^-2^) ‡	113 ± 4	92 ± 3
Glucose (mg/dL)	83 ± 2	86 ± 2
TG (mg/dL)	74 ± 6	82 ± 8
Total cholesterol (mg/dL)	158 ± 4	167 ± 7
HDL cholesterol (mg/dL)	45 ± 2	43 ± 2
VLDL cholesterol (mg/dL)	15 ± 1	17 ± 2
LDL cholesterol (mg/dL)	98 ± 4	108 ± 6

Values are mean **±** SE.

^‡^P < 0.05 significant racial differences.

VO_2peak_−Peak Oxygen consumption; HRmax—maximal heart rat; BMI—body mass index;

TG—triglyceride; HDL—high density lipoprotein; VLDL—very low density lipoprotein; LDL—low density lipoprotein; eGFR—estimated glomerular filtration rate.

### Exercise

Exercise data are shown in Table 2. Both BL and CA experienced significant fluid loss (p<0.05). Fluid loss, ave_HRex, ave_RPEex, water_ex or water_total were not different between control and blockade days. Fluid loss, ave_HRex, ave_RPEex, water_ex or water_total were also not different between CA and BL on either control or blockade days. Ave_HRex in either CA or BL was not different from the 70% HRreserve goal in either condition.

**Table 2:** Exercise hemodynamic and water intake.

	Control		Blockade	
	BL (n = 22)	CA (n = 27)	BL (n = 22)	CA (n = 27)
ave_HRex (bpm)	153 ± 1	152 ± 1	153 ± 1	152 ± 1
ave_RPEex (bpm)	12 ± 0	12 ± 0	12 ± 0	12 ± 0
water_ex (ml)	236 ± 34	311 ± 48	196 ± 27	297 ± 48
water_total (ml)	393 ± 40	498 ± 59	389 ± 27	518 ± 69
Fluid loss (%)	-0.9 ± 0.1 §	-1.0 ± 0.2 §	-0.7 ± 0.2 §	-1.0 ± 0.1 §
Fluid loss (kg)	-0.7 ± 0.1 §	-0.7 ± 0.1 §	-0.6 ± 0.1 §	-0.7 ± 0.1 §

Values are mean **±** SE.

^§^ P < 0.05 significantly different from 0.

ave_HRex—average HR during exercise; ave_RPEex—average RPE during exercise; water_ex—water intake during exercise; water_total—total water intake during study visit; Fluid loss (%)–percent change in body weight from PRE to P90; Fluid loss (kg)–absolute change in body weight from PRE to P90.

### Hemodynamics

**Fig 2:**
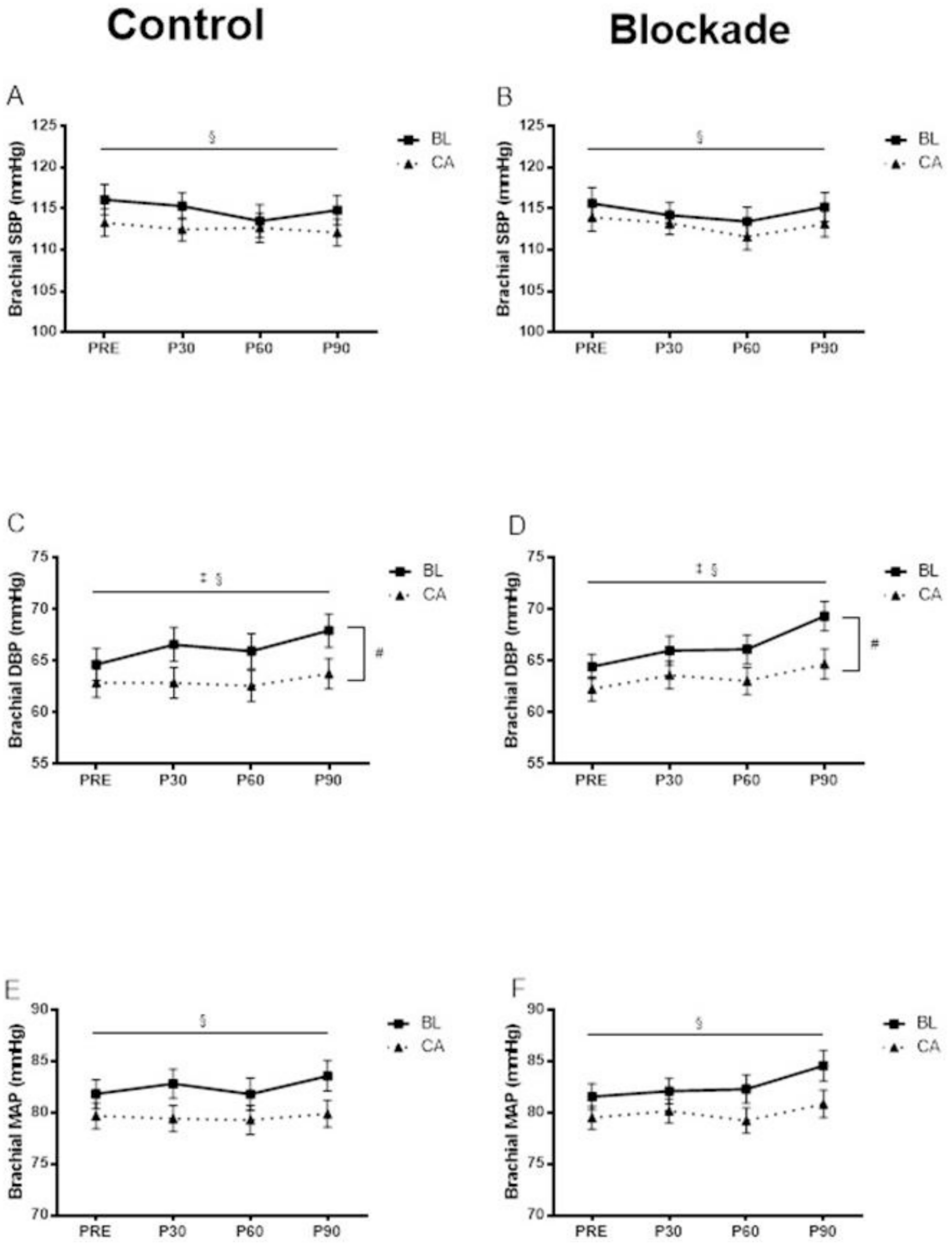
**Brachial SBP at baseline and at 30 min, 60 min and 90 min following aerobic exercise in the control condition (A) and H**_**1**_**R and H**_**2**_**R blockade condition (B).** § Significant main effect of time (p<0.05). **Brachial DBP at baseline and at 30 min, 60 min and 90 min following aerobic exercise in the control condition (C) and H**_**1**_**R and H**_**2**_**R blockade condition (D).** # Significant race by time interaction (p<0.05). * Significant condition by time interaction (p<0.05). § Significant main effect of time (p<0.05). **Brachial MAP at baseline and at 30 min, 60 min and 90 min following aerobic exercise in the control condition (E) and H**_**1**_**R and H**_**2**_**R blockade condition (F).** § Significant main effect of time (p<0.05).

**Fig 3:**
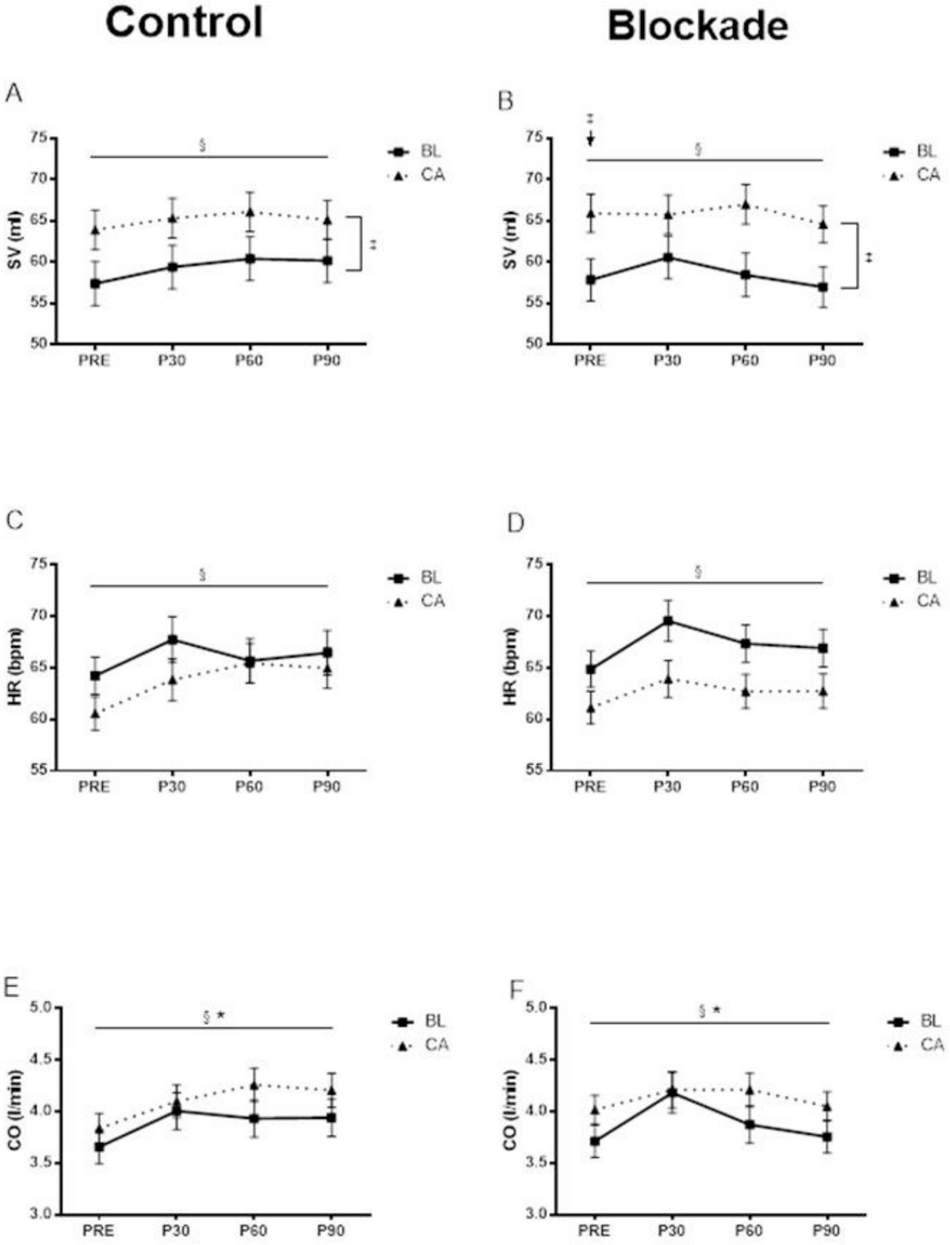
**SV at baseline and at 30 min, 60 min and 90 min following aerobic exercise in the control condition (A) and H**_**1**_**R and H**_**2**_**R blockade condition (B).** § Significant main effect of time (p<0.05). ‡ Significant main effect of race (p<0.05). BL also had significantly lower SV compared to CA at PRE in the blockade condition (p<0.05). **HR at baseline and at 30 min, 60 min and 90 min following aerobic exercise in the control condition (C) and H**_**1**_**R and H**_**2**_**R blockade condition (D).** § Significant main effect of time (p<0.05). **CO at baseline and at 30 min, 60 min and 90 min following aerobic exercise in the control condition (E) and H**_**1**_**R and H**_**2**_**R blockade condition (F).** * Significant condition by time interaction (p<0.05). § Significant main effect of time (p<0.05).

**Fig 4:**
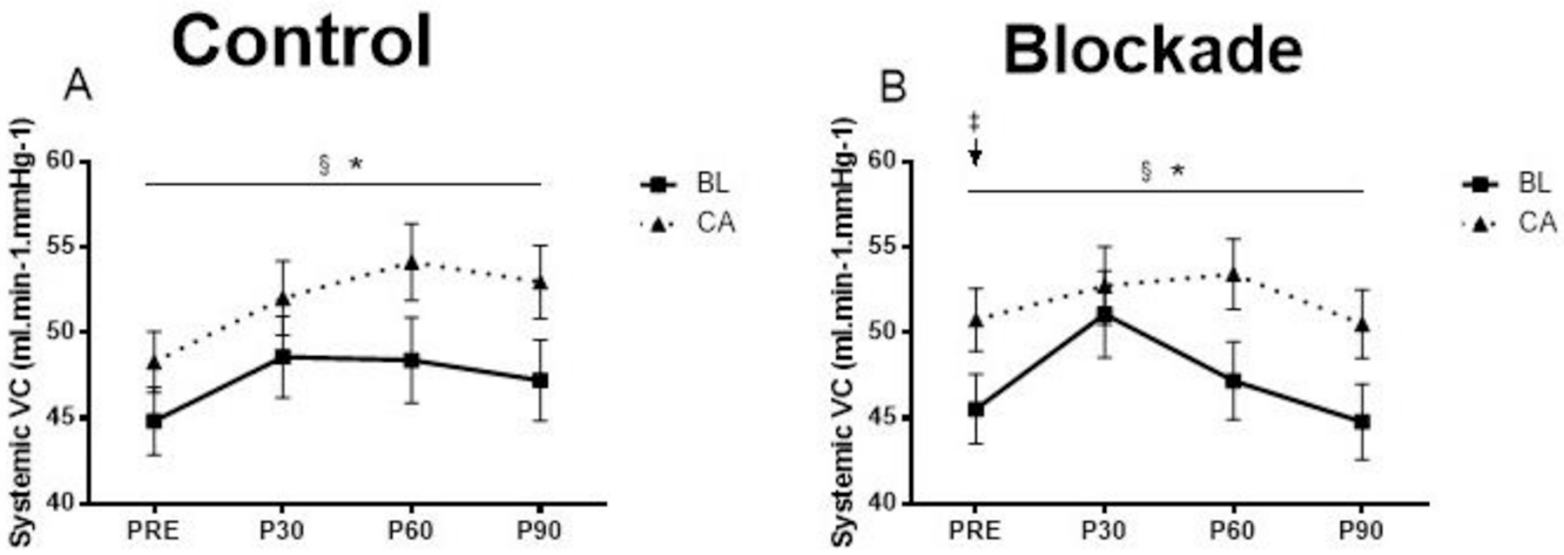
Systemic vascular conductance (SVC) at baseline and at 30 min, 60 min and 90 min following aerobic exercise in the control condition (A) and H_1_R and H_2_R blockade condition (B). * Significant blockade by time interaction. § Significant main effect of time (p<0.05). ‡ BL had significantly lower SVC compared to CA at PRE in the blockade condition (p<0.05).

The hemodynamic measurements at baseline and at 30 min, 60 min and 90 min following acute exercise were shown in Figs 2–4.

### Pre-exercise hemodynamics

Supine resting hemodynamics were not different between control and blockade days. In the control condition, there was no significant difference between BL and CA at rest. However, in blockade condition, BL exhibited significantly lower stroke volume (p<0.05; Fig 3B) and lower systemic vascular conductance (p<0.05; Fig 4D) compared to CA.

### Post-exercise blood pressure

There were no significant interaction effects nor any significant effects of histamine blockade observed for SBP. Overall, Brachial SBP (averaged across group and condition) decreased across time (from 115 ± 1 to 113 ± 1 to 112 ± 1 to 113 ± 1 mmHg, main time effect p<0.05, Fig 2A and 2B).

There was a significant race by time interaction showing that brachial DBP (averaged across condition) increased significantly in BL (from 65 ± 1 to 66 ± 1 to 66 ±1 to 69 ± 2 mmHg) but not in CA (63 ± 1 to 63 ± 1 to 63 ± 1 to 64 ± 1 mmHg) over time (race by time interaction p<0.05,Fig 2C and 2D). There was also a significant condition by time interaction, showing that brachial DBP (averaged across group) increased significantly more in the blockade condition (from 63 ± 1 to 65 ± 1 to 65 ± 1 to 67 ± 1 mmHg) than in the control condition (from 64 ± 1 to 65 ± 1 to 64 ± 1 to 66 ± 1 mmHg) over time (condition by time interaction p<0.05).

There were no significant interaction effects nor any significant effects of histamine blockade for MAP. Overall, Brachial MAP (averaged across group and condition) increased significantly across time (from 81 ± 1 to 81 ± 1 to 81 ± 1 to 82 ± 1 mmHg, time effect p<0.05, Fig 2E and 2F).

### Cardiac parameters

There we no significant interaction effects nor any significant effects of histamine blockade for stroke volume. There was an overall time effect for stroke volume (averaged across group and condition) showing a significant increase across time (from 61 ± 2 to 63 ± 2 to 63 ± 2 to 62 ± 2 ml, time effect p<0.05. Fig 3A and 3B). Stroke volume was also significantly lower (averaged across time and condition) lower in BL compared to CA (59 ± 2 vs. 65 ± 2 ml, race effect p<0.05).

There were no significant interactions nor any effect of histamine blockade on HR. HR (averaged across group and condition) increased significantly across time (from 63 ± 1 to 66 ± 1 to 65 ± 1 to 65 ± 1 ml, time effect p<0.05, Fig 3C and 3D).

Cardiac output (averaged across group) increased significantly less in the blockade condition (from 3.9 ± 0.1 to 4.2 ± 0.1 to 4.0 ± 0.1 to 3.9 ± 0.1 L/min) than in the control condition (from 3.7 ± 0.1 to 4.1 ± 0.1 to 4.1 ± 0.1 to 4.0 ± 0.1 L/min) over time (significant condition by time interaction p<0.05, Fig 3E and 3F). Overall, cardiac output (averaged across group and condition) increased significantly across time (from 3.8 ± 0.1 to 4.1 ± 0.1 to 4.1 ± 0.1 to 4.0 ± 0.1 L/min, significant main effect of time effect p<0.05).

### Vascular conductance

There were no significant changes, no significant group differences or differences due to histamine receptor blockade for femoral vascular conductance. Systemic vascular conductance (averaged across group) increased significantly less in the blockade condition (from 48 ± 1 to 52 ± 2 to 50 ± 2 to 48 ± 1 ml.min-1.mmHg-1) than in the control condition (from 47 ± 1 to 50 ± 2 to 51 ± 2 to 50 ± 2 ml.min-1.mmHg-1) over time (condition by time interaction p<0.05, Fig 4A and 4B). Overall, systemic vascular conductance (averaged across group and condition) increased significantly across time (from 47 ± 1 to 51 ± 1 to 51 ± 1 to 49 ± 1 ml.min-1.mmHg-1, time effect p<0.05).

Exit interviews conducted with subjects at the conclusion of testing showed that the subjects were unable to distinguish between treatment conditions.

## Discussion

This is the first study to examine racial differences in brachial BP following an acute bout of moderate intensity aerobic exercise, with and without histamine blockade, in young normotensive BL and CA men and women. Our major novel findings were as follows: (1) DBP was higher following exercise in BL than in CA without histamine receptor blockade with no differences between groups in cardiac output; (2) There were greater increases in Brachial DBP following exercise during the H1R and H2R blockade condition compared to the control condition. Brachial DBP was also higher in BL compared to CA in the H1R and H2R blockade condition following exercise. Our data show that BL exhibit altered BP control not only at rest (which has been shown in previous studies), but also following exercise which is consistent with an increased risk of hypertension. The BL group in our study was also less fit than CA. However, after adjusting for cardiorespiratory fitness levels, none of the results were altered, suggesting that our findings were not driven by differences in cardiovascular fitness levels, also consistent with prior research in CA [28].

### Responses without histamine blockade

Post-exercise hypotension is well documented in CA, but several studies have reported either unchanged or increased BP following exercise in BL [15, 16]. Enweze et al. [16] found no PEH in young, normotensive BL women 1 hour after cessation of 30 min of moderate-intensity cycling exercise. Furthermore, Pescatello et al. [15] monitored daytime BP by ambulatory BP monitoring after 40 min of moderate-intensity upright cycling exercise. The mean daytime SBP after exercise was increased in both normotensive and hypertensive BL women while no change was found in DBP. In contrast, exercise lowered SBP and DBP in hypertensive CA women but did not have any effect on SBP or DBP in normotensive CA. Thus, prior studies support the notion that BL do not exhibit PEH following moderate intensity endurance exercise. Our data provide support for these findings as brachial DBP increased in BL, but not in CA following exercise. This is also consistent with the notion that a greater stress induced BP response in BL compared to CA, as BL have been shown to exhibit cardiovascular hyper-reactivity to stress with an exaggerated blood pressure response to both behavioral and physiological sympathoexcitation [4].

Post-exercise BP is modulated by changes in peripheral vascular resistance and CO [5, 6]. Central hemodynamics did not contribute to the differential DBP responses, because changes in CO following exercise were not different between BL and CA. Thus, differential changes in peripheral or systemic vascular resistance/conductance likely caused the greater increase in DBP in BL.

Although previous studies have demonstrated significant contribution of the lower limbs in mediating post-exercise BP changes [7, 23, 24], femoral vasodilatation did not seem to contribute to racial differences in BP changes after exercise in our study. We did observe increases in systemic vascular conductance following exercise, but there was no significant increase in femoral vascular conductance in either BL or CA, even though both groups had increased femoral arterial diameter. In contrast to our hypothesis, femoral and systemic vascular conductance were also not different between BL and CA following exercise, and both BL and CA increased systemic vascular conductance. Since vascular conductance was not statistically different between CA and BL in our study, it cannot explain higher DBP in BL subjects. Headley and colleagues [29] suggested that increased systemic vascular resistance in BL and decreased systemic vascular resistance in CA women following exercise was possibly due to higher renin levels and downstream vasoactive substances in middle-aged borderline hypertensive BL women. The discrepancy between their study and ours is probably due to the different subject population. Our subjects were young and normotensive. We also did not measure renin or endothelin-1 levels in our study, but endothelin-1 levels have been shown to be higher in hypertensive BL compared to normotensives of the same race [30]. Therefore, future research in hypertensive BL and CA is warranted.

Although it was suggested that splanchnic, renal or cutaneous circulation has limited contribution to post-exercise BP regulation in CA, their potential contribution to PEH in BL is unknown. Previous research has shown no differences between BL and CA hypertensive patients with respect to the hemodynamic characteristics of the splanchnic or renal circulation [31, 32]. However, BL patients showed a significant positive correlation between the levels of mean arterial pressure and renal vascular resistance while no such correlation was found in CA [31]. In addition, at any level of mean arterial pressure or total peripheral resistance, renal blood flow was significantly less and renal vascular resistance was significantly greater in the BL patients [31]. In our study, both BL and CA had normal kidney function because eGFR in both groups were well above normal values. Therefore, it is less likely that reduced renal blood flow in BL contributes to the increased post-exercise DBP.

Racial difference in DBP was not likely due to differences in hydration status or exercise stimulus because water intake and HR during exercise were not different between CA and BL. Regardless of the cause, the greater increase in DBP in BL is an intriguing finding and deserves follow-up in future studies. Increases in DBP of ~10mmHg or more immediately after exercise (up to 5 min) have been considered an abnormal BP response because it represents an unstable form of hypertension, and may be associated with coronary artery disease (Akhras et al., 1985). The clinical significance of identifying high-risk patients by the increased DBP following exercise in BL should be interpreted with caution because the magnitude of increase in Akhras et al.’s study was greater compared to our study (10~15mmHg vs. 5mmHg) [33]. Therefore, the use of an exercise test as a means of early prediction of hypertension in BL still requires methodological development and confirmation.

Consistent with previous reports, it appears that the decrease in SBP was likely due to increases in Systemic VC despite a small increase in CO [8, 12]. However, the decrease in SBP, although relatively small (~2 mmHg) was significant at 60 min post-exercise. It is unlikely this is a result of an insufficient exercise stimulus because the exercise intensity and duration was shown to consistently elicit PEH in prior studies [6, 12, 34]. The less pronounced PEH in our study may be due to the nature of our young and healthy subject population. This is in agreement with the literature suggesting that the magnitude of PEH is greater in subjects with a higher initial BP level [35, 36] and several studies even suggested that PEH was only observed in young males with hypertension but not in age-matched normotensive males [37, 38].

Surprisingly, we failed to observe decreases in MAP in CA up to 90 min following exercise, which is in contrast to previous studies [24, 28, 39]. In general, 4–5 mmHg decrease in MAP was observed following 60 min of moderate intensity cycling exercise in young normotensive individuals up to 90 min during recovery [24, 28, 39], while there was also evidence of 1.2 mmHg decrease in BP in young and healthy individuals following 120 min of moderate intensity running [40]. The discrepancies in findings between previous studies and ours may be due to the “placebo effect”, a phenomenon often observed in pharmacological studies of hypertension [41]. The subjects in the present study were given identical capsules of either placebo or histamine receptor blockades on two separate study days. The ingestion of novel capsules (even placebos) might elicit sympathoexcitatory responses and elevated BP. Subjects in our study may also exhibit higher cardiovascular reactivity from being exposed to various testing measurements in the lab setting. Although it may appear to be a limitation of our study, it should be noted that cardiovascular reactivity experienced in the lab is correlated with field cardiovascular reactivity [42]. Lastly, a strength of our study was a larger sample size (n = 50) compared to most other studies [24, 28, 39].

### Influence of H1R and H2R antagonists

Although previous studies have demonstrated significant contribution of the histamine receptors in mediating post-exercise BP changes [7, 23, 24], in our study H1R and H2R blockade did not affect post-exercise Brachial SBP or MAP. There were greater increases in Brachial DBP following exercise during the H1R and H2R blockade condition compared to the control condition, possibly driven by diminished increases in SVC, despite a blunted increase in CO in the blockade condition. Brachial DBP was also higher in BL compared to CA in the H1R and H2R blockade condition following exercise.

The vasodilator actions of histamine may be at least partially dependent on nitric oxide (NO) [43]. NO is tonically released from endothelial cells (EC) and is essential to the maintenance of vasodilator tone and homeostasis, which are adversely affected by CVD risk factors [44]. Using invasive techniques, BL have been shown to have reduced NO bioactivity in forearm microcirculation coupled with reduced smooth muscle vasodilator response to NO donors [45]. Forearm blood flow is also significantly attenuated in young BL compared to CA men following various pharmacological infusions including isoproterenol, methacholine, acetylcholine and sodium nitroprusside [46–49]. Additional evidence from in vitro cell culture models performed in human umbilical vein endothelial cells, from BL and CA donors, also support differential responses between race [50]. BL seem to have an EC phenotype characterized by heightened oxidative stress and reduced antioxidant capacity [51]. Considering the notable differences in endothelial-dependent and endothelial-independent vascular dysfunction in BL compared to CA, it is possible that NO plays a crucial role in mediating the differential effects of H1R and H2R antagonists between BL and CA.

Interestingly, baseline differences between BL and CA in stroke volume and systemic vascular conductance were evident after the administration of H1R and H2R blockade but not during the placebo condition. Histamine receptor antagonists do not seem to cause non-specific cardiovascular effects in the absence of exercise stimulus in CA. Intravenous infusion of H1R (hydroxyzine) or H2R (cimetidine) antagonist or a combination of both antagonists did not alter HR or BP in CA subjects [52]. Oral ingestion of a H1R antagonist (540 mg of fexofenadine) or H2R antagonist (300 mg of ranitidine) also did not change CO, HR, BP, leg blood flow or skin blood flow under normal resting conditions [23, 24]. The mechanism responsible for lower stroke volume and systemic vascular conductance at rest in BL after H1R and H2R blockade in our current study at is not clear. It is possible that BL are more responsive to H1R and H2R blockade compared to CA. Infusion of histamine has been shown to increase cardiac contractility as percentage cardiac fractional shortening increased from 38.2 ± 4.1 to 53.5 ± 3.6% and mean fiber shortening velocity increased from 1.31 ± 0.19 to 1.99 ± 0.22 cm/s. These changes were both greatly reduced by H2R blockade along with H1R blockade [53]. BL may have decreased cardiac contractility in response to the H1R and H2R blockades even at rest without infusion of additional histamine and the hyper-responsiveness to H1R and H2R blockade remained during post-exercise recovery. This may explain the observed lower stroke volume in BL compared to CA during recovery from exercise during H1R and H2R blockaded trial in our study.

### Limitations

We acknowledge potential limitations of the present study. We were unable to identity the countries of origin in our participants of blacks and Caucasians and there may be diversity within the descent populations potentially associated with disperse genetic influences. Despite this limitation, our study showed racial differences in cardiovascular responses following acute exercise and histamine blockade.

In conclusion, an acute bout of aerobic exercise provokes an increase in DBP during the post-exercise period in young African-Americans, but not in persons of European descent. Moreover, DBP is also elevated in BL after exercise with histamine receptor blockade. However, H1R and H2R blockade elicited differential responses in cardiac function between BL and CA following exercise, suggesting a potential role of histamine receptors in mediating post-exercise BP in BL. The heightened BP and vascular responses to exercise stimulus is consistent with the greater CVD risk in BL.
